# Dexmedetomidine inhibits inflammatory response and autophagy through the circLrp1b/miR-27a-3p/Dram2 pathway in a rat model of traumatic brain injury

**DOI:** 10.18632/aging.103975

**Published:** 2020-11-04

**Authors:** Hengchang Li, Chengxiang Lu, Wenfei Yao, Lixin Xu, Jun Zhou, Bin Zheng

**Affiliations:** 1 Department of Anesthesiology, Guangzhou First People's Hospital, School of Medicine, South China University of Technology, Guangzhou, Guangdong, China; 2 Department of Anesthesiology, The Third Affiliated Hospital of Southern Medical University, Guangzhou, Guangdong, China

**Keywords:** dexmedetomidine, traumatic brain injury, autophagy, circLrp1b, inflammatory response

## Abstract

Circular RNAs (circRNAs) have a regulatory function on inflammation and autophagy, of which rno-circRNA_010705 (circLrp1b) appears to be significantly up-regulated following traumatic brain injury (TBI). Dexmedetomidine (DEX) shows improvement effects in TBI by inhibiting NLRP3/caspase-1. However, whether circLrp1b plays critical roles in DEX-mediated TBI attenuation and the underlying mechanisms remain unclear. After TBI was established in rats by controlled cortical impact (CCI) to cause brain trauma, they received an intracerebroventricular injection of lentiviral vector, followed by intraperitoneal injection of DEX. Administration of DEX ameliorated autophagy in rats following TBI, accompanied by up-regulated circLrp1b and Dram2 and down-regulated miR-27a-3p. DEX promoted the effects of circLrp1b in attenuating TBI-induced neurologic impairment, autophagy, and inflammation, which was significantly reversed by inhibition of miR-27a-3p or Dram2 overexpression. Mechanistically, northern blot and luciferase reporter assays indicated that circLrp1b up-regulated Dram2 expression by functioning as a sponge for miR-27a-3p to promote autophagy involved in TBI, which was reversed by DEX treatment. Collectively, this study demonstrated that DEX inhibits inflammatory response and autophagy involved in TBI *in vivo* through inactivation of the circLrp1b/miR-27a-3p/Dram2 signaling pathway.

## INTRODUCTION

Severe traumatic brain injury (TBI) is characterized by axon destruction, neuron loss, and demyelination. The injury poses serious medical, public health, and societal burden [1, 2]. Primary TBI caused by direct physical damage to the brain tissue from an external impact cannot be usually reversed. Secondary TBI is the subsequent biochemical changes, including inflammation, apoptosis, exacerbated neuronal loss, and autophagy, and can be reversed in most cases [3, 4]. Among these post-traumatic secondary pathological responses, persistently activated autophagy after TBI is considered to cause deterioration of nerve injury [5–8]. On the contrary, several reports indicate that enhancement of autophagy could attenuate TBI-induced secondary neuronal death [9–11]. Therefore, modulation of TBI-induced neuronal autophagy has therapeutic implications.

Dexmedetomidine (DEX), a highly selective α-2-adrenergic receptor agonist, is a new type of clinical anesthetic widely applied in surgical procedures for its ability to rapidly shorten postoperative cognitive impairments [12–14]. Accumulating evidence demonstrates that DEX has the potential to prevent various types of organ injuries, including brain ischemia, acute lung injury [15, 16], acute kidney injury [17], and myocardial ischemia [18]. Several recent studies have demonstrated that DEX exerts its neuroprotective effects through the inactivation of pro-inflammatory signaling pathways [19, 20]. In agreement with this notion, our previous study further revealed that the neuroprotective effect of DEX in TBI is associated with inhibition of NLRP3 inflammasome formation/caspase-1 expression [21]. Interestingly, DEX-induced modulation of autophagy has drawn attention to the adrenergic receptor agonist's protective role in various injuries. For example, Luo et al. [22] reported that post-conditioning with DEX at the beginning of reperfusion protected mouse brain from ischemia-reperfusion injury by inhibiting neuronal autophagy. Ding et al. [23] demonstrated that DEX effectively reversed lipopolysaccharide-induced acute lung injury by reducing inflammation and autophagy. Zhu et al. pointed out that post-conditioning with DEX could improve learning and memory dysfunction in cerebral ischemia–reperfusion injury and reduce inflammation and autophagy [24]. However, whether or not the improvement effect of DEX in TBI is realized via regulation of autophagy, as well as the underlying molecular mechanisms, remains unclear.

Circular RNA (circRNA), a new type of endogenous noncoding RNA, is characterized by conservation, stability, abundance, and specificity [25]. Emerging evidence reveals that circRNAs regulate multiple biological functions in different diseases. They realize this regulation by functioning as a competitive endogenous RNA to modify downstream genes by sponging miRNAs [26–28]. To the best of our knowledge, the biological function of circRNAs in TBI has been rarely reported, except for some bioinformatics analysis based on the alteration of circRNA expression in rats after TBI. Xie et al. [29] used circRNA microarrays to profile the altered circRNAs in rat hippocampus after TBI and validated four differentially expressed circRNAs. Among the four circRNAs, the up-regulated rno-circRNA_010705 (circLrp1b) drew our attention for its interaction with miR-27a-3p. Considering that miR-27a is down-regulated in the injured cortex after TBI, contributing to increase in pro-apoptotic proteins and thereby neuronal death [30], and that DNA damage regulated autophagy modulator 2 (*Dram2*) is a predicted target gene of miR-27a-3p, we proposed that the circLrp1b/miR-27a-3p/Dram2 axis plays an important role in DEX-mediated modulation of autophagy in TBI.

To verify this hypothesis, we first established a TBI rat model and focused on studying the effects of DEX on the expression levels of circLrp1b, miR-27a-3p, and Dram2. Next, we evaluated the biological functions of circLrp1b, miR-27a-3p, and Dram2 in the neurological outcome, autophagy, and inflammation. Subsequently, we assessed the synergistic effect of DEX and circLrp1b, miR-27a-3p, and Dram2 on TBI to assess whether circLrp1b positively regulates Dram2 by functioning as a sponge for miR-27a-3p in TBI. Overall, our findings will further uncover whether the improved neurological outcome is associated with DEX-attenuated circLrp1b/miR-27a-3p/Dram2-mediated autophagy in the hippocampus.

## RESULTS

### DEX reverses TBI-mediated regulation of circLrp1b, miR-27a-3p, and Dram2, as well as autophagy, in rats

**Figure 1:**
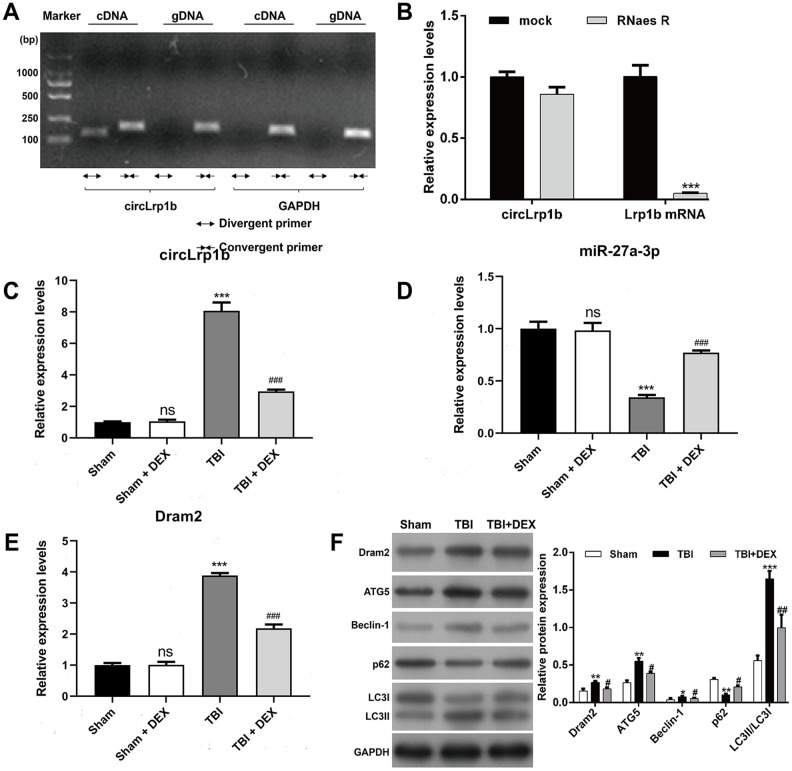
**Expression levels of circLrp1b, miR-27a-3p, Dram2, and autophagy-associated molecules in traumatic brain injury rats treated with dexmedetomidine.** (**A**) circLrp1b in traumatic brain injury (TBI) brain tissues. Divergent primers detect circular RNAs in cDNA, but not in gDNA. GAPDH served as a negative control. (**B**) Abundance of circLrp1b and Lrp1b mRNA, as determined using quantitative real-time reverse transcriptase polymerase chain reaction (qRT-PCR), in injured brain tissues treated with or without RNase R; cDNA, complementary DNA; gDNA, genomic DNA. ***p < 0.001, compared with mock. After the rats were subjected to TBI, intraperitoneal injection of 20 μg/kg dexmedetomidine (DEX) was administered for 4 consecutive days. Expression levels of circLrp1b (**C**), miR-27a-3p, (**D**) and Dram2 (**E**) in Sham, Sham + DEX, TBI, and TBI + DEX groups, as determined using qRT-PCR. Each experiment was repeated 6 times. **p < 0.01, ***p < 0.001; ns, not significant, compared with Sham; #*p* < 0.05, ##*p* < 0.01, ###*p* < 0.001, compared with TBI. (**F**) Expressions of the Dram2, ATG5, Beclin-1, p62, and LC3 I/II proteins, as measured by western blot.

PCR using divergent primers was performed to confirm the circular structure of circLrp1b. As is shown in Figure 1A, only the circular transcripts of Lrp1b were amplified by divergent primers in cDNA, not in gDNA. Meanwhile, we observed that no product was amplified by divergent primers of *GAPDH* (negative control gene in cDNA and gDNA). RNase R assay was performed to further confirm the stability of circLrp1b. The findings demonstrated that the linear transcripts of Lrp1b were obviously degraded by RNase R, whereas the circular transcripts of Lrp1b harbors a loop structure with resistance to RNase R treatment (Figure 1B). Altogether, the data confirmed the stability of the circular structure of circLrp1b.

To investigate whether circLrp1b, miR-27a-3p, and Dram2 are associated with DEX-induced effects in TBI, we analyzed their expression levels in a TBI rat model treated with DEX. Results from qRT-PCR analysis revealed that TBI caused significant up-regulation of circLrp1b (Figure 1C) and down-regulation of miR-27a-3p (Figure 1D), which were reversed by DEX administration. In addition, we found that the expression of Dram2 mRNA and protein was significantly elevated in the TBI group, but remarkably reduced after DEX treatment (Figure 1E, 1F). However, DEX treatment did not affect the expression levels of circLrp1b, miR-27a-3p, or Dram2 in the sham group. Considering the association between autophagy and TBI, we additionally measured several proteins related to autophagy using western blot. As is depicted in Figure 1F, TBI induction up-regulated the expression levels of ATG5, Beclin-1, and LC3 I/II and down-regulated the expression level of p62; all of these effects were attenuated following DEX treatment. These data indicate that circLrp1b and miR-27a-3p play essential roles in DEX-induced effects in TBI rats.

### Down-regulation of circLrp1b enhances DEX-induced neurological outcomes in TBI rats

**Figure 2:**
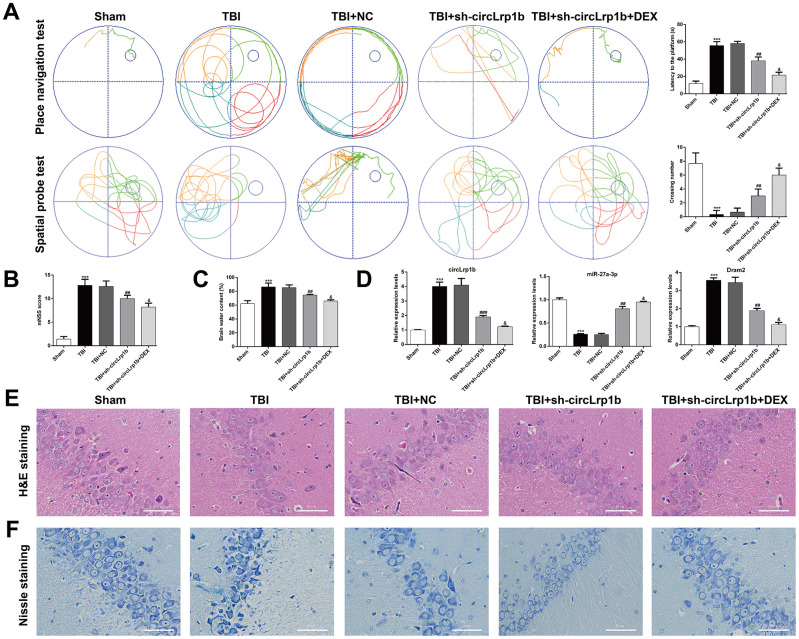
**Down-regulation of circLrp1b in rat brain enhances the effects of dexmedetomidine in neurological outcome after traumatic brain injury.** Rats were administered an intracerebroventricular injection of sh-circLrp1b before TBI induction, followed by intraperitoneal injection of 20 μg/kg dexmedetomidine (DEX). (**A**) Representative track plots of animal paths in the place navigation task and spatial probe task of the Morris water maze test in rats from different groups (left panel) and quantification of the latency time and the number of the crossing were recorded (right panel). (**B**) Application of the modified Neurological Severity Score (mNSS). (**C**) Calculation of the brain water content, as is described in the Materials and Methods section. (**D**) Expression levels of circLrp1b, miR-27a-3p, and Dram2, as determined using real-time quantitative reverse transcriptase polymerase chain reaction (qRT-PCR). Each experiment was repeated 6 times. ***p < 0.001, compared with Sham; ##*p* < 0.01, ###*p* < 0.001, compared with TBI + NC; &*p* < 0.05, compared with TBI + sh-circLrp1b. Representative 400× images of the hematoxylin and eosin (H&E)-stained (**E**) and Nissl-stained (**F**) hippocampal sections from the experimental groups. Scale bar: 50 μm.

The significant increase in the circLrp1b expression prompted us to analyze whether circLrp1b inhibition enhances the protective effects of DEX in TBI-induced neurological dysfunctions. First, the Morris water maze test was performed to evaluate spatial learning and memory abilities after TBI. As is shown in Figure 2A, TBI induced a longer latency to find the platform and reduced the time to cross the platform zone, which was abolished by down-regulation of circLrp1b and further reversed by co-treatment with sh-circLrp1b and DEX. Second, we evaluated the effects of sh-circLrp1b in TBI-induced brain damage. We found that the affected rats demonstrated severe neurological impairment, as was indicated by increased mNSS score (Figure 2B) and apparent edema (Figure 2C) due to increased brain water content. Both these effects were decreased after treatment with sh-circLrp1b and further decreased significantly after treatment with a combination of sh-circLrp1b and DEX. Meanwhile, the expression levels of circLrp1b and Dram2 were down-regulated in TBI rats, whereas that of miR-27a-3p was up-regulated in circLrp1b-knockdown TBI rats. Both of these effects were enhanced by additional DEX treatment (Figure 2D). Finally, we assessed the effects of sh-circLrp1b in TBI-induced hippocampal neuron damage. H&E staining (Figure 2E) revealed that the hippocampal neurons in the Sham group were intact and had normal morphology, in which the cells have round nuclei, prominent nucleoli, clear cytoplasm, regular structure, and higher density. However, TBI induction modified this morphology, as reflected by irregular cell contour, edema, low density, and loose chromatin. The affected morphological changes were ameliorated by prior treatment with sh-circLrp1b alone or DEX plus sh-circLrp1b. Similarly, Nissl staining of the TBI tissue revealed pronounced neuronal loss, which could also be mitigated by prior treatment with sh-circLrp1b alone or DEX plus sh-circLrp1b (Figure 2F).

### Down-regulation of circLrp1b enhances DEX-mediated suppression of TBI-induced autophagy and inflammation

**Figure 3:**
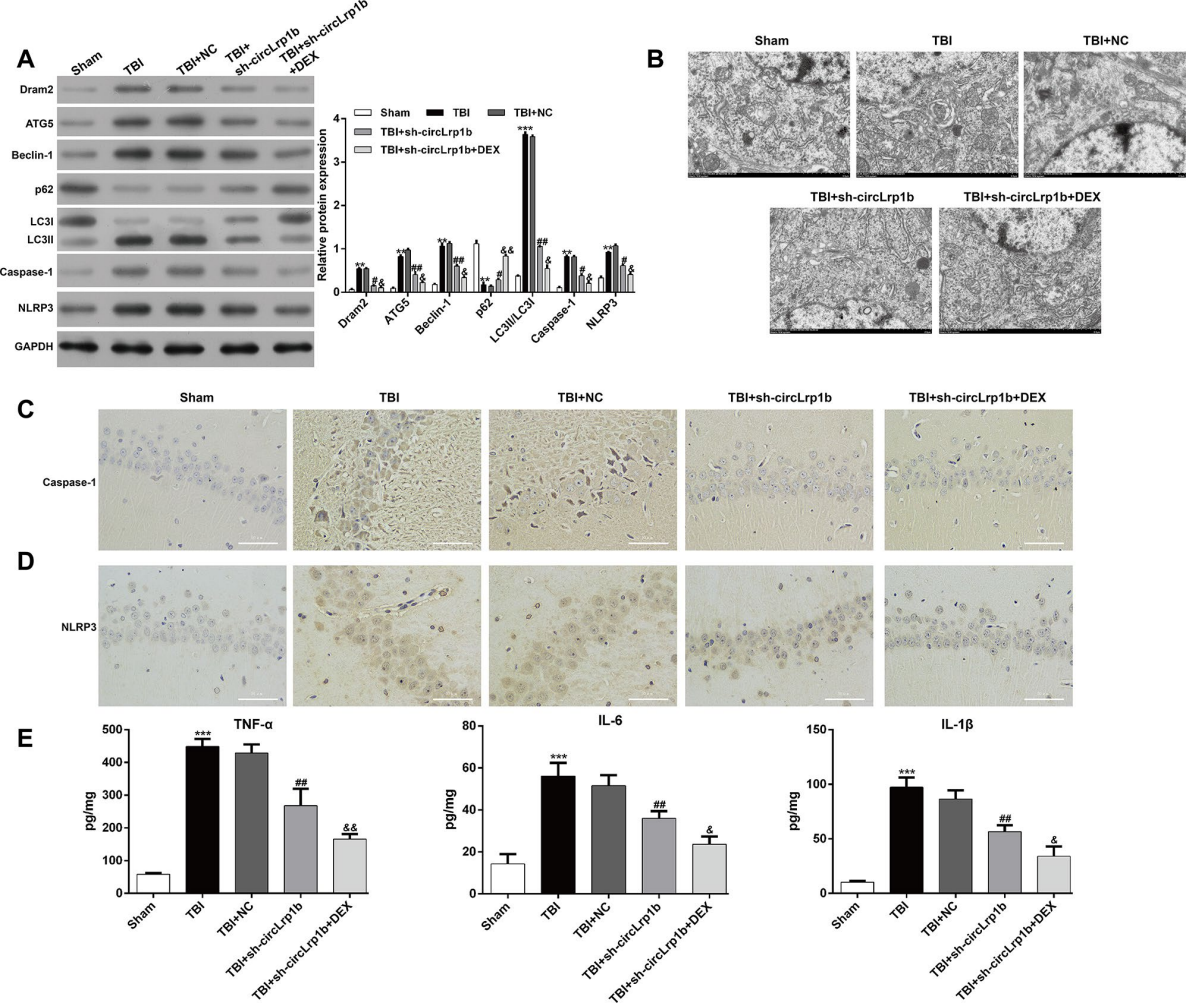
**Down-regulation of circLrp1b enhances the effects of dexmedetomidine in reducing traumatic brain injury-induced autophagy and inflammation.** Rats were administered an intracerebroventricular injection of sh-circLrp1b before traumatic brain injury (TBI) induction, followed by intraperitoneal injection of 20 μg/kg dexmedetomidine (DEX). (**A**) Expression levels of Dram2, ATG5, Beclin-1, p62, LC3 I/II, caspase-1, and NLRP3 proteins, as measured by western blot. (**B**) Representative electron microscopic images of autophagosomes of hippocampal tissues obtained from different animal groups. The images of the immunohistochemical staining of caspase-1 (**C**) and NLRP3 (**D**) are presented. Scale bar: 50 μm. (**E**) Quantitative analysis of enzyme-linked immunosorbent assay (ELISA) detection of TNF-α, IL-6, and IL-1β production in the hippocampal tissues. Each experiment was repeated 6 times. **p < 0.01, ***p < 0.001, compared with Sham; #*p* < 0.05, ##*p* < 0.01, compared with TBI + NC; &*p* < 0.05, &&*p* < 0.01, compared with TBI + sh-circLrp1b.

In molecular analysis, we examined the effects of circLrp1b knockdown on autophagy-related protein expression. As is shown in Figure 3A, down-regulation of circLrp1b obviously down-regulated the autophagy proteins Dram2, ATG5, Beclin-1, LC3 I, and LC3 II, but up-regulated p62. Moreover, in the hippocampal tissues derived from TBI rats, the expression levels of caspase-1 and NLRP3 were decreased, which was further strengthened by DEX treatment. Next, transmission electron microscopic analysis of the hippocampal tissues revealed the accumulation of autophagosomes in the TBI group compared with the sham group. This accumulation was notably decreased after circLrp1b knockdown and further decreased with DEX treatment (Figure 3B). Consistent with the western blot findings, immunohistochemical staining confirmed significantly elevated expressions of the inflammasomes caspase-1 (Figure 3C) and NLRP3 (Figure 3D) in TBI rats. This elevation was impaired after treatment with sh-circLrp1b with or without DEX. Moreover, the production of inflammatory cytokines, including TNF-α, IL-6, and IL-1β, in the hippocampal tissues was first induced by TBI surgery. This induction was suppressed by treatment with sh-circLrp1b and further suppressed by co-administration of sh-circLrp1b and DEX (Figure 3E).

### miR-27a-3p inhibition reverses the effects of circLrp1b knockdown in TBI-induced autophagy and inflammation

**Figure 4:**
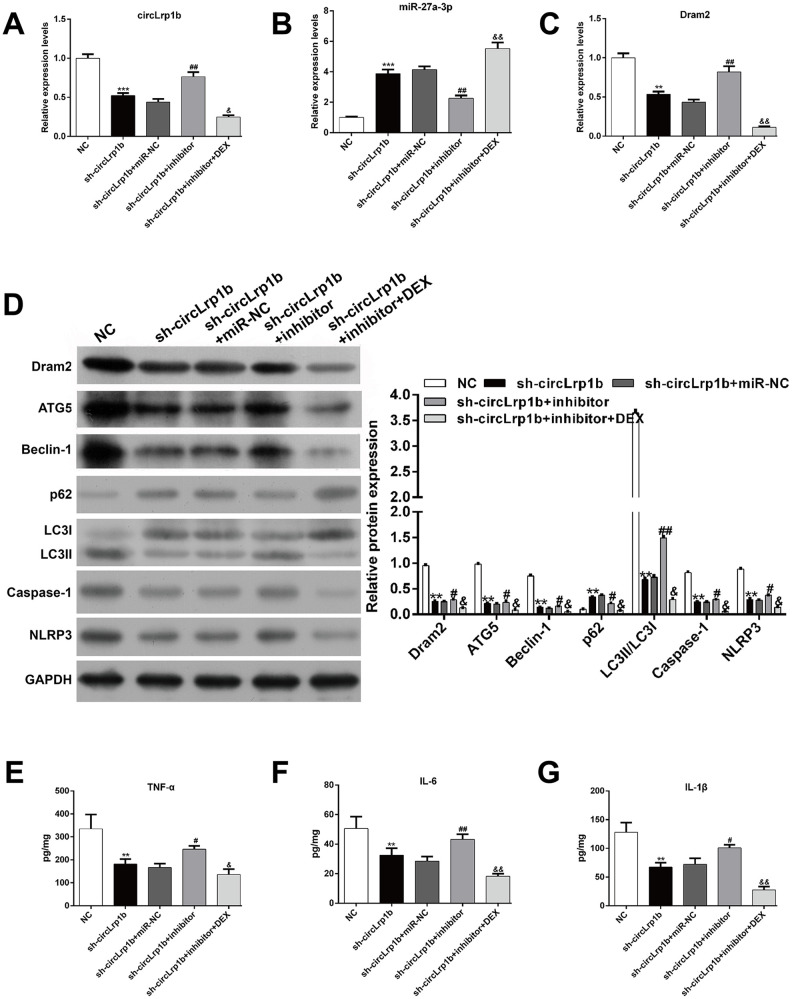
**Inhibition of miR-27a-3p reverses the effects of circLrp1b knockdown in traumatic brain injury-induced autophagy and inflammation.** Rats were administered intracerebroventricular injection of lentivirus vectors of sh-circLrp1b and miR-27a-3p inhibitor before traumatic brain injury (TBI) induction, followed by intraperitoneal injection of 20 μg/kg dexmedetomidine (DEX). Expression levels of circLrp1b (**A**), miR-27a-3p (**B**), and Dram2 (**C**), as determined using real-time quantitative reverse transcriptase polymerase chain reaction. (**D**) Expression levels of the proteins Dram2, ATG5, Beclin-1, p62, LC3 I/II, caspase-1, and NLRP3, as measured by western blot. Quantitative analysis of TNF-α (**E**), IL-6 (**F**), and IL-1β (**G**) production in the hippocampal tissues by using enzyme-linked immunosorbent assay (ELISA). Each experiment was repeated 6 times. **p < 0.01, ***p < 0.001, compared with NC; #*p* < 0.05, ##*p* < 0.01, compared with sh-circLrp1b + miR-NC; &*p* < 0.05, &&*p* < 0.01, compared with sh-circLrp1b + inhibitor.

We next explored via rescue experiment whether decrease in miR-27a-3p was crucial for TBI-induced autophagy, apoptosis, and inflammation. Briefly, TBI-induced rats received intracerebroventricular injection of lentivirus vectors of sh-circLrp1b and miR-27a-3p inhibitors before TBI induction, followed by intraperitoneal injection of DEX. We first confirmed that, in circLrp1b-knockdown TBI rats, inhibition of miR-27a-3p reversed the mRNA expressions of circLrp1b (Figure 4A), miR-27a-3p (Figure 4B), and Dram2 (Figure 4C), which were weakened by DEX treatment. As was expected, inhibition of miR-27a-3p impaired the effects of sh-circLrp1b on the expression of TBI-induced autophagy and inflammatory proteins, and these effects were reversed by DEX treatment (Figure 4D). In addition, ELISA for TNF-α (Figure 4E), IL-6 (Figure 4F), and IL-1β (Figure 4G) yielded similar results. These data indicate that circLrp1b and miR-27a-3p possibly present the opposite effects in TBI-induced autophagy, apoptosis, and inflammation.

### Dram2 restoration abolishes the effects of circLrp1b knockdown in TBI-induced neurological outcome, autophagy, and inflammation

**Figure 5:**
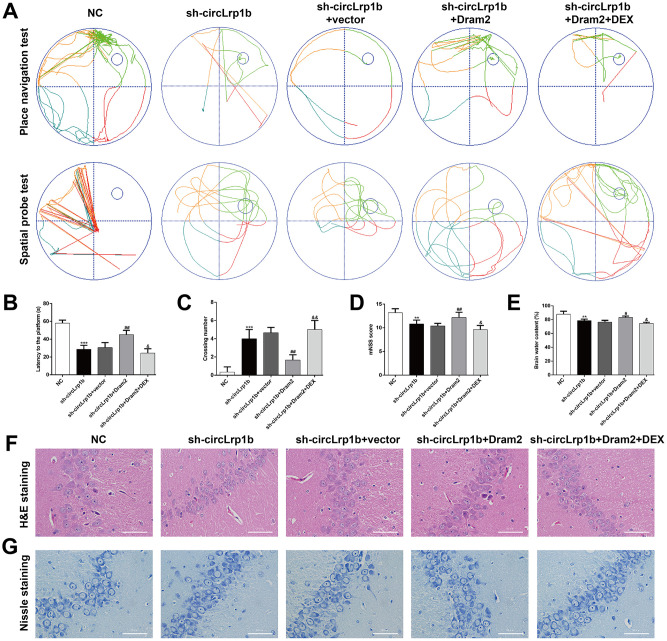
**Restoration of Dram2 abolishes the effects of circLrp1b knockdown in traumatic brain injury-induced neurological outcome.** Rats were administered intracerebroventricular injection of lentivirus vectors of sh-circLrp1b and Dram2 before traumatic brain injury induction, followed by intraperitoneal injection of 20 μg/kg dexmedetomidine (DEX). (**A**) Representative track plots of animal paths in the place navigation task and spatial probe task of the Morris water maze test in rats from different groups. (**B**, **C**) Quantification of the latency time and the number of crossing were recorded. (**D**) Analysis of the modified Neurological Severity Score (mNSS). (**E**) Calculation of the brain water content was calculated as described in the Materials and Methods section. Each experiment was repeated 6 times. **p < 0.01, ***p < 0.001, compared with NC; #*p* < 0.05, ##*p* < 0.01, compared with sh-circLrp1b + vector; &*p* < 0.05, &&*p* < 0.01, compared with sh-circLrp1b + Dram2. Representative 400× images of the hematoxylin and eosin (H&E)-stained (**F**) and Nissl-stained (**G**) hippocampal sections obtained from the experimental groups. Scale bar: 50 μm.

**Figure 6:**
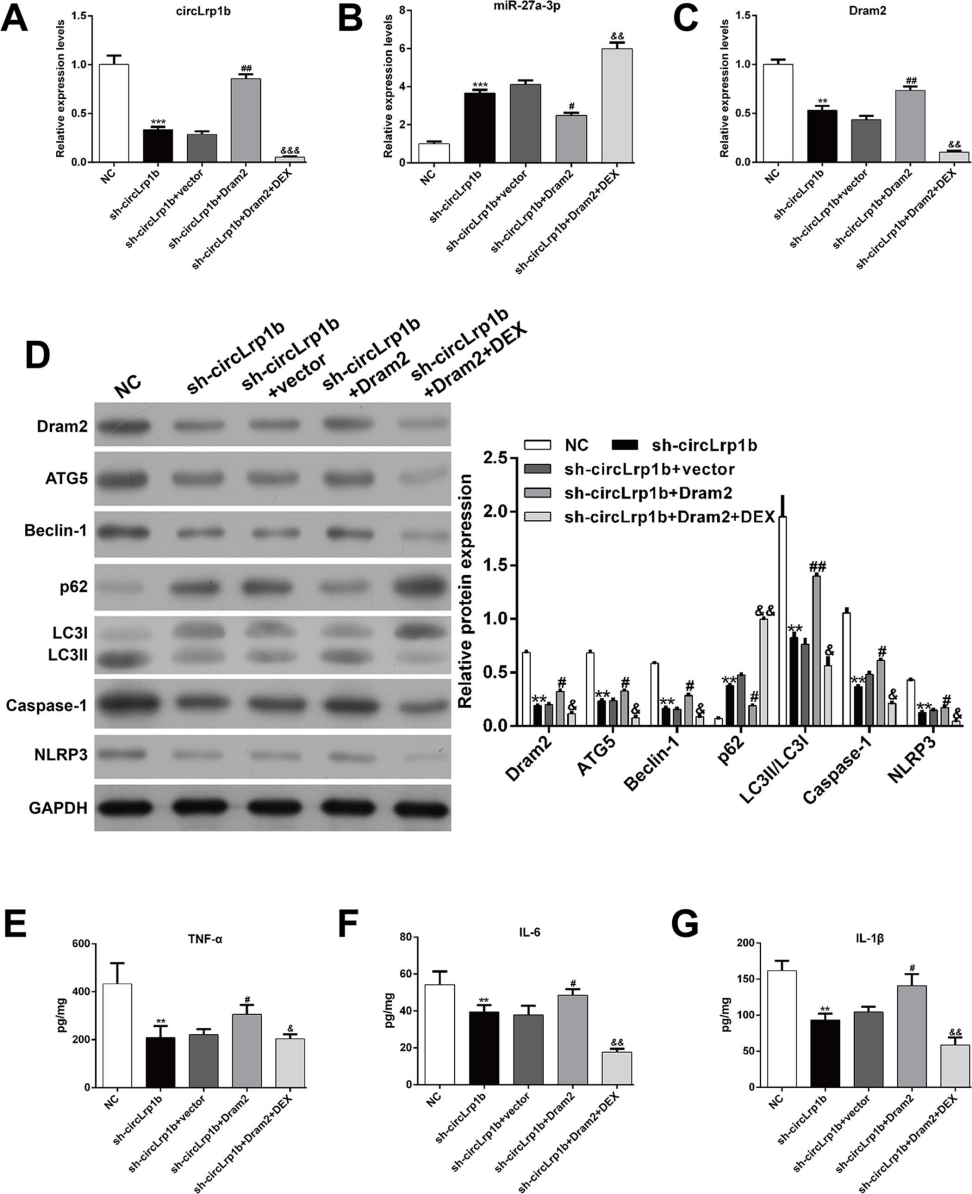
**Restoration of Dram2 abolishes the effects of circLrp1b knockdown in traumatic brain injury-induced autophagy and inflammation.** Rats were administered intracerebroventricular injection of lentivirus vectors of sh-circLrp1b and Dram2 before traumatic brain injury (TBI) induction, followed by intraperitoneal injection of 20 μg/kg dexmedetomidine (DEX). The expression levels of circLrp1b (**A**), miR-27a-3p (**B**), and Dram2 (**C**), as determined using real-time quantitative reverse transcriptase polymerase chain reaction. (**D**) The expression levels of Dram2, ATG5, Beclin-1, p62, LC3 I/II, caspase-1, and NLRP3 proteins, as evaluated using western blot. Quantitative analysis of TNF-α (**E**), IL-6 (**F**), and IL-1β (**G**) production in the hippocampal tissues by using enzyme-linked immunosorbent assay (ELISA). Each experiment was repeated 6 times. **p < 0.01, ***p < 0.001, compared with NC; #*p* < 0.05, ##*p* < 0.01, compared with sh-circLrp1b + vector; &*p* < 0.05, &&*p* < 0.01, &&&*p* < 0.001, compared with sh-circLrp1b + Dram2.

Dram2, which is an autophagy protein, was up-regulated in TBI rats. Therefore, we proposed that DEX-mediated down-regulation of Dram2 could be another mechanism underlying the agonist's neuroprotective effects in TBI. To validate this, rats were administered an intracerebroventricular injection of lentivirus vectors of sh-circLrp1b and Dram2 before TBI induction, followed by intraperitoneal injection of DEX. The Morris water maze test revealed that overexpression of Dram2 significantly reversed the improvement effects of circLrp1b knockdown in TBI-induced spatial learning and memory impairment. This effect was notably abolished after DEX treatment (Figure 5A–5C). Moreover, mNSS score (Figure 5D) and the brain water content test (Figure 5E) consistently indicated that Dram2 overexpression aggravated the attenuated neurological impairment caused by circLrp1b knockdown and that this effect was reversed considerably by DEX treatment. H&E (Figure 5F) and Nissl (Figure 5G) staining further demonstrated that reduced TBI-induced hippocampal neuron damage caused by sh-circLrp1b was obviously reversed by Dram2 overexpression with or without DEX treatment. Similar to the effects of miR-27a-3p inhibition, Dram2 overexpression reversed the mRNA expressions of circLrp1b (Figure 6A), miR-27a-3p (Figure 6B), and Dram2 (Figure 6C) caused by circLrp1b knockdown in TBI rats, and these effects were weakened by DEX treatment. Furthermore, the suppressive effects of sh-circLrp1b in TBI-induced autophagy and inflammation were reversed by restoring Dram2 expression, as was demonstrated by western blot analysis (Figure 6D) and ELISA (Figure 6E–6G). These data indicate that circLrp1b and Dram2 possibly exert synergic effects in TBI-induced neurological outcome, autophagy, and inflammation.

### Dram2 knockdown enhances the suppressive effects of miR-27a-3p in TBI-induced neurological outcome, autophagy, and inflammation

**Figure 7:**
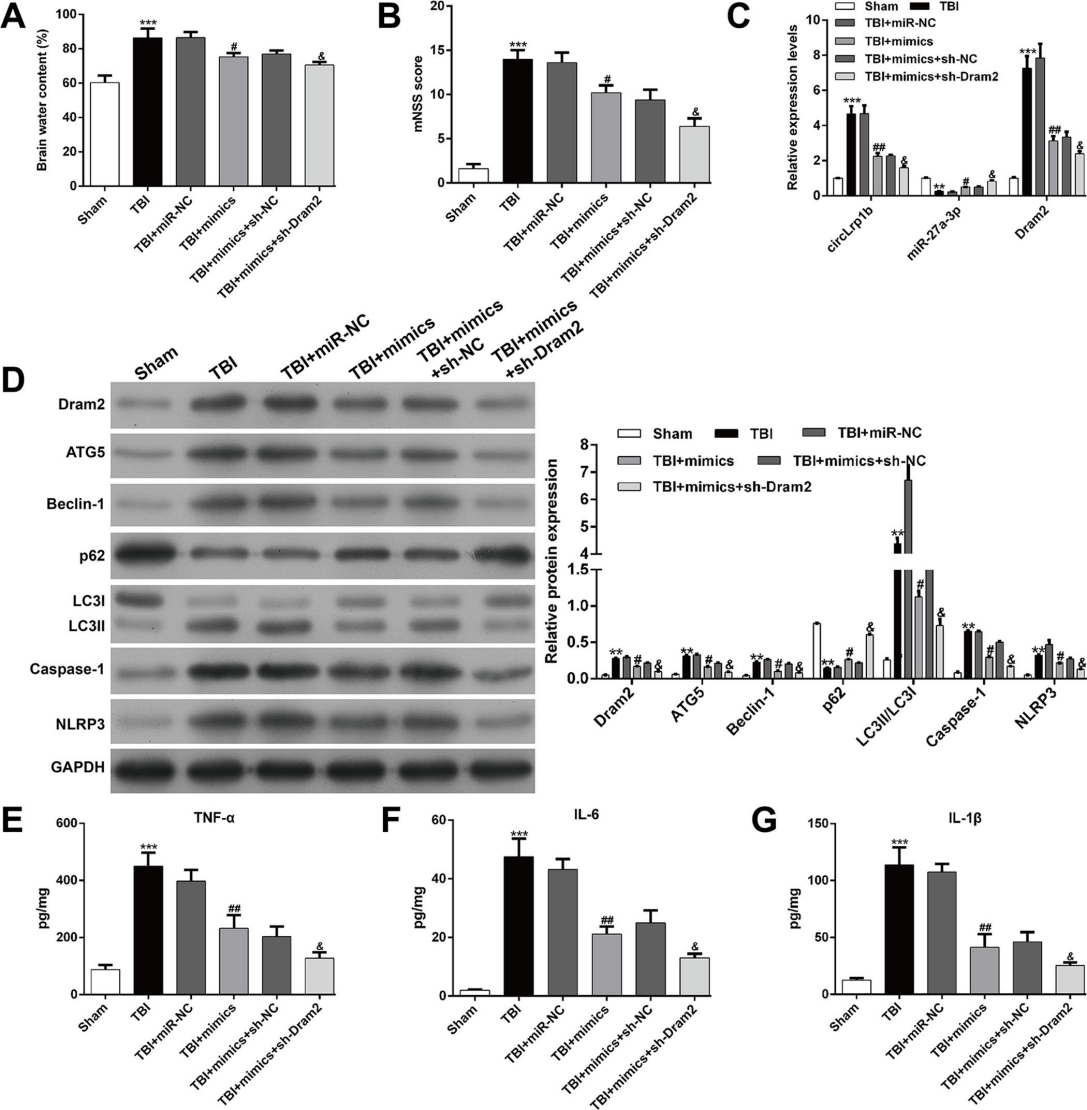
**Knockdown of Dram2 enhances the suppressive effects of miR-27a-3p in traumatic brain injury-induced neurological outcome, autophagy, and inflammation.** Rats were administered intracerebroventricular injection of lentivirus vectors of miR-27a-3p mimics and sh-Dram2 before traumatic brain injury (TBI) induction. (**A**) Calculation of the brain water content, as described in the Materials and Methods section. (**B**) Analysis of the modified Neurological Severity Score (mNSS). (**C**) Expression levels of circLrp1b, miR-27a-3p, and Dram2, as determined by real-time quantitative reverse transcriptase polymerase chain reaction. (**D**) Expression levels of Dram2, ATG5, Beclin-1, p62, LC3 I/II, caspase-1, and NLRP3 proteins, as measured using western blot. Quantitative analysis of enzyme-linked immunosorbent assay (ELISA) detection of TNF-α (**E**), IL-6 (**F**), and IL-1β (**G**) production in the hippocampal tissues. Each experiment was repeated 6 times. **p < 0.01, ***p < 0.001, compared with Sham; #*p* < 0.05, ##*p* < 0.01, compared with TBI + miR-NC; &*p* < 0.05, compared with TBI + mimics + sh-NC.

To further confirm whether or not Dram2 is a downstream regulator in miR-27a-3p in TBI-induced neurological outcome, autophagy, and inflammation, rats were pretreated with an intracerebroventricular injection of lentivirus vectors of miR-27a-3p mimics and sh-Dram2. We found that miR-27a-3p overexpression significantly reversed the brain water content (Figure 7A) and mNSS score (Figure 7B) in rats after TBI, which was further strengthened by a combination of miR-27a-3p overexpression and Dram2 knockdown. PCR analysis revealed that miR-27a-3p overexpression decreased the expression of circLrp1b and Dram2 and increased the expression of miR-27a-3p in TBI rats. These effects were enhanced by a combination of miR-27a-3p overexpression and Dram2 knockdown (Figure 7C). Moreover, the TBI-induced autophagy proteins Dram2, ATG5, Beclin-1 and LC3 I/II and the inflammasomes caspase-1 and NLRP3 were attenuated by miR-27a-3p overexpression and further impaired by Dram2 knockdown (Figure 7D), but the opposite effects were noted for P62. In addition, ELISA for TNF-α (Figure 7E), IL-6 (Figure 7F), and IL-1β (Figure 7G) yielded similar results.

### CircLrp1b acts as a sponge for miR-27a-3p, and Dram2 is a direct target of miR-27a-3p

**Figure 8:**
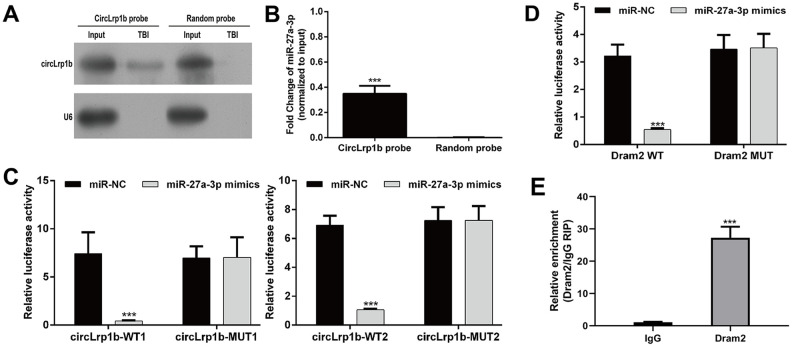
**CircLrp1b acts as a sponge for miR-27a-3p, and Dram2 is a direct target of miR-27a-3p.** (**A**) Endogenous circLrp1b expression in brain tissues derived from traumatic brain injury (TBI) rats, validated by northern blot. (**B**) Quantification of northern blot; ***p < 0.001, compared with random probe. (**C**) Luciferase reporter assays demonstrating miR-27a-3p as a direct target of circLrp1b. (**D**) Luciferase reporter assays demonstrating Dram2 as a direct target of miR-27a-3p. (**E**) RNA-binding protein immunoprecipitation (RIP) assay revealing the interaction of miR-27a-3p and Dram2. Each experiment was repeated 6 times. ***p < 0.001, compared with miR-NC; data are expressed as mean ± standard deviation (SD).

On the basis of the aforementioned findings, we speculated that circLrp1b/miR-27a-3p/Dram2 could be an important signaling pathway involved in DEX-induced neuroprotective effects in TBI rats. To validate this notion, we performed the following experiments. First, the TBI brain tissues were subjected to northern blot analysis. Second, the overexpression of circLrp1b in brain tissues (Figure 8A) and specific enrichment of miR-27a-3p was validated by using biotinylated probes against circLrp1b (Figure 8B). Third, by using RNA 22v2, we discovered that circLrp1b and miR-27a-3p had two complementary base sequences. Through constructing the corresponding luciferase reporter constructs, we found that the up-regulation of miR-27a-3p significantly decreased the luciferase activities of the two WT constructs for circLrp1b, but not of the MUT constructs (Figure 8C), supporting the direct interaction of circLrp1b and miR-27a-3p. Similarly, the complementary sequences of miR-27a-3p and Dram2 were predicted using TargetScan. Using the luciferase reporter assay (Figure 8D), we further demonstrated that the luciferase activity of the constructs carrying the WT Dram2 3'-UTR was significantly reduced, whereas that carrying the MUT Dram2 3'-UTR did not change following overexpression of miR-27a-3p, indicating Dram as a direct target of miR-27a-3p. Furthermore, the RIP assay revealed that miR-27a-3p interacted with Dram2 (Figure 8E), further confirming that *Dram2* is a target gene of miR-27a-3p. Altogether, these results suggest that circLrp1b can regulate Dram2 expression by functioning as a sponge for miR-27a-3p in TBI.

## DISCUSSION

DEX has been widely used in patients receiving surgery for its critical role in protecting the central nervous system and improving overall neurological outcomes of surgical procedures [31]. In addition to its anesthetic function, recent studies have been studying the potential of DEX in brain protection against multiple damages [32, 33]. DEX exerts its neuroprotective effects in TBI through multiple mechanisms, including anti-inflammation [21], anti-apoptosis [34], and anti-oxidative stress [35]. Similarly, the suppressive effects of DEX in the inflammatory response against TBI have been reported by the following studies. For example, Ding et al. [36] demonstrated that DEX reduced the production of TNF-α, IL-6, IL-8, and IL-1β. Zheng et al. [21] also pointed out that DEX could inhibit NLRP3 in the rat hippocampus after TBI. In the present study, we found the inhibition of autophagy by DEX treatment was accompanied by the improved neurological outcome and reduced inflammation and apoptosis in hippocampal tissues derived from TBI rats. Consistent with this finding, Luo et al. reported that DEX mediated its neuroprotective effects after cerebral ischemia by reducing neuronal autophagy via the up-regulation of HIF-1α [22]. Shen et al. [37] also demonstrated that DEX-mediated activation of the PI3K/AKT/mTOR signaling pathway alleviates the degree of TBI by suppressing the activation of neuronic autophagy. However, some studies have presented evidence that autophagic responses to TBI are protective and beneficial after injury [9–11]. These inconsistencies could be attributed to differences in the study context and mechanisms leading to perturbation of autophagy.

Recently, circRNAs have been reported to play central roles in regulating autophagy proteins, which are potential therapeutic targets in treating various diseases, including myocardial infarction [38], cerebral ischemic stroke [39], and cancer [40, 41]. However, the functional role of circRNAs in TBI remains unclear. Xie et al. [29] successfully identified the alteration of circRNA expression in the hippocampus after TBI and predicted the potential binding of up-regulated rno-circRNA_010705 (circLrp1b) and miR-27a-3p by using bioinformatics analysis and circRNA/miRNA interaction prediction. On the basis of this finding, we further explored whether circLrp1b and miR-27a-3p are involved in the DEX-mediated neuroprotection in TBI. As was expected, the up-regulated circLrp1b and down-regulated miR-27a-3p in the TBI rat hippocampus were reversed by DEX treatment. By conducting loss-of-function assays and rescue experiments, we found that down-regulation of circLrp1b improved the neurological outcome after TBI and reduced TBI-induced autophagy and inflammation and that these effects were notably abolished by miR-27a-3p inhibition. Similarly, miR-27a was rapidly down-regulated in TBI rat brains, whereas overexpression of miR-27a effectively attenuated brain damage [7, 30]. Moreover, miR-27a-3p appears to have protective effects in blood-brain barrier disruption and brain injury via inhibition of neuronal apoptosis and microglia activation [42]. Zhang et al. reported that miR-27a exerts a cardioprotective effect in hypoxia-induced H9C2 cell injury by regulating autophagy and apoptosis via targeting of ATG7 [43]. Other studies have also demonstrated the anti-inflammatory effects of miR-27a in spinal cord injury [44], osteoarthritis [45], and pancreatitis [46]. These findings undoubtedly demonstrate that circLrp1b and miR-27a-3p play opposite roles in TBI-induced neurological outcome, autophagy, apoptosis, and inflammation.

Moreover, we demonstrated that circLrp1b has a circular structure, circLrp1b acts as a sponge for miR-27a-3p, and Dram2 is a novel target of miR-27a-3p. Furthermore, restoration of Dram2 abolished the effects of circLrp1b knockdown in TBI-induced neurological outcome, autophagy, and inflammation. There is accumulating evidence of circRNAs serving as miRNA sponges, which affect the posttranscriptional actions of miRNAs as suppressors of the translation and/or stability of target mRNAs [47–49]. The autophagy protein Dram2 seems to play an oncogenic role in non-small cell lung cancer via p53 regulation [50], which is a target gene of miR-144 in mycobacterium tuberculosis [51] and miR-125a in retinoblastoma [52]. These observations suggest that inhibition of autophagy is involved in the DEX-mediated neuroprotective effect and that the DEX activity is possibly facilitated by the circLrp1b/miR-27a-3p/Dram2 signaling pathway.

**Figure 9:**
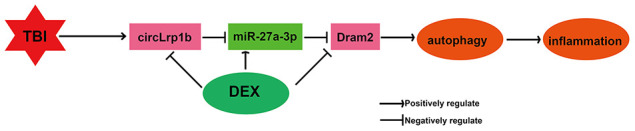
**Illustration of a hypothetical model.** Dexmedetomidine inhibits traumatic brain injury-induced inflammatory response and autophagy *in vivo* through inactivation of the circLrp1b/miR-27a-3p/Dram2 signaling pathway.

In summary, the present study demonstrated for the first time that DEX inhibits inflammation and autophagy in TBI *in vivo* through inactivation of the circLrp1b/miR-27a-3p/Dram2 signaling pathway (Figure 9). Our findings provide new insights into the molecular mechanisms underlying DEX-mediated neuroprotection in TBI, which will assist in looking for new therapeutic candidates.

## MATERIALS AND METHODS

### Establishment of the TBI rat model

Adult male Sprague-Dawley (SD) rats (age, 12–16 weeks; body weight, 280–300 g) were purchased from the Experimental Animal Center of Southern Medical University (Guangzhou, China) and housed under standard conditions (i.e., temperature, 22 ± 2 °C; light-dark cycle, 12–12 h; relative humidity, 45%–75%) with free access to food and water. TBI was induced by controlled cortical impact (CCI) to cause brain trauma, as is previously described [21]. Briefly, the rats were anesthetized by intraperitoneal injection of 1% pentobarbital sodium (50 mg/kg). They were then placed in a stereotactic frame to receive craniotomy, in which a 6-mm cranial window was created on the right side midway between the bregma and lambda for receiving CCI injury in the right hemisphere. The sham group rats received identical surgical procedures except for the CCI injury. All procedures were approved by the ethics committee of the Guangzhou First People's Hospital and strictly performed in accordance with the guidelines on animal care of the Guangzhou First People's Hospital.

### Experimental groups

Lentivirus vectors of circLrp1b (sh-circLrp1b) and corresponding negative control (NC) were purchased from RiboBio Co., Ltd. (Guangzhou, China). Lentivirus vectors of miR-27a-3p inhibitor, miR-27a-3p mimics, miR-NC, Dram2 overexpression plasmid (pcDNA3.1-Dram2), empty vector, sh-Dram2, and sh-NC were designed and synthesized by VipotionBio Co., Ltd. (Guangzhou, China). DEX was provided by Jiangsu Heng Rui Medicine Co., Ltd (Jiangsu, China). All rats were randomly allocated to different groups on the basis of the following protocols. For Protocol I, SD rats were randomly allocated to four groups (n = 10 per group), which were Sham, Sham + DEX, TBI, and TBI + DEX (rats were subjected to CCI, followed by intraperitoneal injection of 20 μg/kg DEX for 4 consecutive days). The sham group was administrated the same volume of saline or DEX.

For Protocol II, SD rats were randomly allocated to 13 groups (n = 10 per group): TBI + NC; TBI + sh-circLrp1b; TBI + sh-circLrp1b + DEX; sh-circLrp1b + miR-NC; sh-circLrp1b + inhibitor; sh-circLrp1b + inhibitor + DEX; sh-circLrp1b + vector; sh-circLrp1b + Dram2; sh-circLrp1b + Dram2 + DEX; TBI + miR-NC; TBI + mimics; TBI + mimics + sh-NC; and TBI + mimics + sh-Dram2. Briefly, the groups in Protocol II received CCI induction, in which the corresponding oligonucleotide was administered by intracerebroventricular injection of approximately 1 × 10^7^ transfection unit lentivirus vectors of sh-circLrp1b, inhibitor, or Dram2 in a total fluid volume of 10 μL for 5 days before CCI induction, followed by intraperitoneal injection of 20 μg/kg DEX for 4 consecutive days.

### Animal treatments

All rats in the above groups were fed for 7 days after the last drug administration. On day 8, rats received the Morris water maze test. On day 9, the rats received modified neurological severity scoring (mNSS). Next, rats from different groups were killed by an overdose of CO_2_. The collected brain tissues were stored at −80 °C or treated with paraformaldehyde or glutaraldehyde.

### Morris water maze test

The hippocampus-dependent spatial learning and memory was evaluated by two investigators blind to the experimental design, according to the previous reports [53, 54]. Briefly, all rats underwent the place navigation task, which involves searching a visible platform placed at the center of a quadrant (target quadrant) in 60 s. The escape latency to reach the platform was recorded. For the spatial probe test, the platform was removed and the rats were placed in the quadrant opposite from the target quadrant and forced to swim for 60 s. The number of times the rats crossed the target quadrant was measured. The track images in place navigation and spatial probe tests were captured using ANY-maze software (USA).

### Neurobehavioral evaluation

The mNSS [55] is an 18-point scale (0, normal; 18, maximal deficit) to evaluate the neurological outcome. Higher scores indicate severe neurobehavioral impairment.

### Brain water content measurement

The wet–dry method was used to measure the brain water content, according to a previous protocol [56]. Briefly, after rats from different groups were administered CO_2_ euthanasia, the wet weight of the brain was recorded. After the brains were dried in a desiccating oven, their dry weights were recorded. The brain water content was calculated by the following formula: (wet weight − dry weight)/wet weight × 100%.

### Real-time quantitative reverse transcriptase PCR

Total RNA was extracted from the hippocampal tissues of rats by using TRIzol reagent (Invitrogen, CA, USA), and cDNA was synthesized using a qRT-PCR kit (Bestar; DBI Bioscience). qRT-PCR was performed in a Stratagene RT-PCR system (Applied Biosystems, USA) by using SYBR^®^ Green PCR Master Mix (DBI Bioscience). The thermal cycling conditions were as follows: 95 °C for 2 min, followed by 40 cycles of 94 °C for 20 s, 58 °C for 20 s, and 72 °C for 40 s. The relative expression of each target gene was quantified using the 2^−ΔΔCt^ method. GAPDH was used to normalize circLrp1b and Dram2 mRNA. U6 served as an endogenous control for miR-27a-3p. The primer sequences are listed in Table 1.

**Table 1:** Primer sequences used for PCR analysis.

**Gene**	**Primer sequence**
rno_circRNA_010705-Divergent	F: 5‘-AACTGCAAACCACAGACCT-3’
	R: 5′-GCCTGGCTGACACTTAAAT-3’
rno_circRNA_010705-Convergent	F: 5‘-AGCTGCTCTCCCGACTATTT-3’
	RT: 5′-ATCGCCAGTGGTCTGGAATA-3’
rno-miR-27a-3p	F: 5‘-ACACTCCAGCTGGGTTCACAGTGGCTAAGTT-3’
	RT: 5′-CTCAACTGGTGTCGTGGAGTCGGCAATTCAGTTGAGGCGGAACT-3’
Dram2	F: 5‘-TATCGCTGCCGTTTTAGGCA-3’
	R: 5′-ATCCCAAGTACAAGGCCAGC-3’
GAPDH	F: 5‘-CCTCGTCTCATAGACAAGATGGT-3’
	R: 5′-GGGTAGAGTCATACTGGAACATG-3’
U6	F: 5‘-CTCGCTTCGGCAGCACA-3’
	R: 5′-AACGCTTCACGAATTTGCGT-3’

F, forward; R: reverse

### Western blot

Total protein was extracted from the hippocampal tissues obtained from the rats using an RIPA buffer (Solarbio Life Sciences, Beijing, China). Protein concentration was measured using a commercial BCA kit (Pierce, Rockford, IL, USA). Next, the protein samples were separated by 10% SDS-PAGE and transferred to a polyvinylidene fluoride membrane. After blocking with 5% fat-free milk, the membrane was incubated overnight at 4 °C with primary antibodies of Dram2 (1:1000, ab230191), ATG5 (1:1000, ab228525), Beclin-1 (1:1000, ab228525), p62 (1:1000, ab56416), LC3 I/II (1:1000, ab48394), caspase-1 (1:1000, ab62698), NLRP3 (1:1000, ab214185), and GAPDH (1:5000, ab8245). Subsequently, the membrane was incubated with secondary horseradish peroxidase (HRP)-conjugated immunoglobulin G (IgG) antibodies (1:5000) for 2 h at 37 °C, followed by detection of protein bands using enhanced chemiluminescence (ECL).

### Histochemical examination

After CO_2_ euthanasia, the whole brain obtained from different groups was fixed in 4% paraformaldehyde, paraffin-embedded, and cut into 4-μm thick sections. The sections were subjected to hematoxylin and eosin (H&E) staining, Toluidine blue (Nissl) staining, and immunohistochemical staining for caspase-1 (1:1000, ab62698) and NLRP3 (1:1000, ab214185), according to standard protocols. The staining images were captured under a light microscope (DM2500; Leica Microsystems, Germany) at 400× magnification.

### Enzyme-linked immunosorbent assay

The concentrations (pg/mg) of TNF-α, IL-6, and IL-1β in the hippocampal tissues were determined using corresponding ELISA kits, as directed by the manufacturer.

### Transmission electron microscopy

The autophagy level was assessed by transmission electron microscopic analysis. Briefly, the hippocampal tissues were fixed at 4°C in 2.5% glutaraldehyde, post fixed in 2% osmium tetroxide, embedded in epoxy resin, and sectioned with an ultramicrotome. The thin sections were stained with 4% uranyl acetate and 2.5% lead nitrate. The autophagosomes were observed and imaged using a transmission electron microscope (TEM; JEM-1200; Jeol Ltd, Tokyo, Japan).

### Confirmation of the circular structure of circLrp1b

PCR using divergent primers and RNase R treatment were performed to confirm the circular structure of circLrp1b. For PCR using divergent primers, genomic DNA (gDNA) was extracted from brain tissues of TBI rats by employing a PureLink™ Genomic DNA Mini Kit (Thermo Fisher Scientific, USA). The circular and linear transcripts of Lrp1b in both cDNA and gDNA from the injured brain tissues were amplified by divergent and convergent primers, respectively. Next, the results were verified by 2% agarose gel electrophoresis. GAPDH served as the negative control. For RNase R treatment, total RNA (10 μg) extracted from the injured brain tissues was mixed with 40 U of RNAse R for 2 h at 37 °C. The expression levels and stability of circLrp1b and line Lrp1b mRNA were determined using qRT-PCR.

### Northern blot

Total RNA was isolated from the brain tissues of TBI rats by using TRIzol reagent (Invitrogen, CA, USA). Meanwhile, the circLrp1b probe for northern blot was achieved using the PCR DIG Probe Synthesis Kit (Roche, Mannheim, Germany). After the RNA samples were denatured in formaldehyde, they were separated using 1% agarose-formaldehyde gel electrophoresis and transferred to nylon membranes (GE Healthcare, Buckinghamshire, UK). The membranes were cross-linked and incubated overnight with s circLrp1b probe or random probe. RNA signals were visualized using Chemiluminescent Detection Buffer (Thermo Scientific).

### Target prediction

The potential miR-27a-3p–binding sites of circLrp1b were predicted using RegRNA 2.0. The potential Dram2-binding sites of miR-27a-3p were predicted using TargetScan (http://www.targetscan.org).

### Luciferase reporter assay

For luciferase reporter assay, the constructs containing two wild-type (circLrp1b-WT1 and circLrp1b-WT2) or the corresponding two mutant circLrp1b (circLrp1b-MUT1 and circLrp1b-MUT2) in the miR-27a-3p–binding sites, as well as Dram2 WT and Dram2 MUT in the miR-27a-3p–binding sites, were generated using the psiCHECK2 dual luciferase vector (Promega, Madison, WI, USA). Subsequently, the aforementioned constructs (40 ng) was transfected to 293 T cells together with miR-27a-3p mimics/miR-NC (10 pmol) for 48 h using Lipofectamine^®^ 2000 (Invitrogen). Thereafter, firefly and Renilla luciferase activities were determined using the Dual-Luciferase Reporter Assay Kit (Promega). Luciferase activity was measured using the Dual Luciferase Assay System (Promega). The data were normalized to the firefly luciferase signal.

### RNA-binding protein immunoprecipitation assay

RNA-binding protein immunoprecipitation (RIP) assay was conducted using an RNA-binding protein immunoprecipitation kit (Magna; Millipore, Billerica, MA, USA), according to the manufacturer's instructions. Briefly, brain tissue lysates were incubated with the RIP buffer containing magnetic beads that were conjugated with anti-Dram2 antibody (Abcam, Cambridge, UK) or negative control IgG (Abcam). After incubation with proteinase K, the immunoprecipitated RNA was isolated and analyzed using qRT-PCR to demonstrate the binding of miR-27a-3p.

### Statistical analysis

Each quantitative experiment was performed 6 times. All continuous data were analyzed using SPSS 20.0 (SPSS, Inc., Chicago, IL, USA) and expressed as mean ± standard deviation (SD). One-way ANOVA, followed by Tukey post hoc test or Student t-test, was used to evaluate significant differences. A p-value of <0.05 was considered statistically significant.

### Ethics approval

All procedures were strictly performed in accordance with the guidelines on animal care of the Guangzhou First People's Hospital (Guangzhou, China) and approved by the ethics committee of the Guangzhou First People's Hospital.
